# Longitudinal relationships between anxiety and fear of progression in post-CABG patients: a cross-sectional and cross-lagged panel network analysis

**DOI:** 10.3389/fpsyt.2026.1937200

**Published:** 2026-10-14

**Authors:** Zhengwei Luo, Fenghui Chen, Huihui Li, Weilong Wang, Duohuan Chen, Yongzhong Guo, Juan Xing, Fang Liang, Jianshuang Tao

**Affiliations:** 1 Department of Nursing, Xinjiang Medical University, Urumqi, China; 2 Department of Cardiac Surgery, People’s Hospital of Xinjiang Uygur Autonomous Region, Urumqi, China; 3 Department of Cardiac Surgery, First Affiliated Hospital of Xinjiang Medical University, Urumqi, China

**Keywords:** anxiety, coronary artery bypass grafting, cross-lagged panel network analysis, fear of progression, network analysis

## Abstract

**Background:**

Patients following coronary artery bypass grafting (CABG) frequently experience elevated anxiety and fear of progression (FoP), which has a high comorbidity and significantly increases adverse postoperative event risks. This prospective study explored the dynamic longitudinal associations between anxiety and FoP in post-CABG patients using network analysis methods.

**Methods:**

A prospective longitudinal design enrolled first-time CABG patients from three tertiary hospitals in Urumqi, China. Anxiety and FoP were assessed using the Generalized Anxiety Disorder-7 (GAD-7) and the Fear of Progression Questionnaire-Short Form (FoP-Q-SF) at three time points: hospital discharge (T1), 1 month (T2), and 3 months (T3) post-discharge. Cross-sectional Gaussian Graphical Models were constructed at each time point, and cross-lagged panel network (CLPN) analysis was performed to examine longitudinal symptom associations. Six *a priori* selected covariates (age, gender, educational level, duration of CHD, chronic comorbidities, and NYHA functional classification) were residualized prior to network estimation.

**Results:**

A total of 343 patients were included in the network analysis. From T1 to T3, total GAD-7 scores demonstrated a notable downward trend, whereas FoP-Q-SF scores exhibited a continuous upward trend. The core node at both T1 and T2 was FoP11 “Worrying about what will become of the family if something should happen to the patient, “ which shifted to FoP2 “Being nervous prior to doctors’ appointments or periodic examinations” at T3. In the T1-T2 CLPN, the strongest cross-lagged edge was from GAD7 “Feeling afraid as if something awful might happen” to FoP2 “Being nervous prior to doctors’ appointments or periodic examinations”. In the T2-T3 CLPN, the strongest cross-lagged edge was from GAD2 “Not being able to stop or control worrying” to FoP6 “Being afraid of the possibility that the children could contract disease”. In the T1-T3 network, the strongest cross-lagged edge was from GAD4 “Trouble relaxing” to FoP3 “Being afraid of pain”. Anxiety nodes generally exhibited higher Out-Expected Influence, while FoP nodes showed higher In-Expected Influence.

**Conclusion:**

Anxiety levels gradually decreased during early rehabilitation, whereas fear of progression increased over time. Early anxiety symptoms showed positive cross-lagged associations with later fear of progression. These findings suggest that clinical nurses should consider phased, precision-based psychological interventions targeting stage-specific symptoms during different rehabilitation stages.

## 1 Introduction

According to the China Cardiovascular Health and Disease Report 2023, coronary heart disease (CHD) is the leading contributor to the burden of cardiovascular disease in China, with an estimated 11.39 million prevalent cases, and its mortality rate has been continuously rising since 2012 (1). Coronary artery bypass grafting (CABG) remains the preferred revascularization strategy for complex coronary artery lesions, with the annual number of procedures performed in China reaching 49, 000 in 2023 (1). Patients following CABG frequently experience anxiety symptoms, which may be attributed to factors such as surgical trauma, intraoperative near-death experiences, and postoperative pain. Studies have reported that the incidence of perioperative anxiety in CABG patients can be as high as 22% (2). Furthermore, the long-term prognosis of these patients remains challenging. It has been reported that the 10-year all-cause mortality rate can reach 28% (3), and the 30-day readmission rate is 14% (4). Confronted with the need for long-term medication and the risk of recurrent cardiovascular events, patients often bear a substantial psychological burden due to persistent worries about disease progression (5).

Fear of Progression (FoP) refers to an individual’s concern about disease symptoms and apprehension about potential disease exacerbation, loss of control, or life-threatening complications (6). Research indicates that moderate levels of FoP may prompt patients to pay closer attention to their health, improve treatment adherence and self-management capabilities (7), and encourage active participation in rehabilitation (5). However, prolonged and excessive fear may not only trigger psychological issues such as anxiety and depression, impairing physical and mental health (8), but also increase readmission rates and exacerbate the economic burden on both families and society (6). Notably, FoP and anxiety exhibit a high degree of comorbidity and a positive correlation. Their interaction not only diminishes patients’ quality of life and adherence but also increases the risk of postoperative adverse events, leading to delayed wound healing, higher readmission rates, and increased mortality (9). Therefore, effectively managing anxiety and fear among CABG patients is a critical step toward improving the quality of their recovery.

Although previous studies have confirmed a close correlation between anxiety and FoP in patients following CABG (6), most existing literature relies on cross-sectional designs, which only reveal static associations between symptoms and fail to elucidate the temporal relationships and underlying directional longitudinal associations between anxiety and FoP. Furthermore, traditional correlation and regression analyses are insufficient for identifying core nodes within symptom clusters or their dynamic evolutionary patterns, thereby limiting the discovery of targets for precision psychological interventions. In recent years, network analysis, as a cutting-edge psychological methodology, has enabled precise assessment of complex relationships between symptoms through visualization models, facilitating the identification of core symptoms within symptom clusters (10). Building upon this, the cross-lagged panel network (CLPN) extends network analysis to longitudinal data, enabling the simultaneous examination of cross-temporal directional longitudinal associations and dynamic evolution processes among all symptoms within the network model (11).

In view of this, the current network analysis study aims to conduct a prospective longitudinal study with two primary objectives: First, to identify the core nodes and dynamic evolutionary characteristics of the anxiety and FoP symptom networks at different time points following CABG (day of discharge, 1 month post-discharge, and 3 months post-discharge) using cross-sectional network analysis; Second, to explore the longitudinal associations between anxiety and FoP symptoms via CLPN analysis. This study aims to provide empirical evidence to inform the development of phased, precision-based psychological intervention strategies for nursing staff.

## 2 Methods

### 2.1 Participants

This study employed a prospective longitudinal design. Using a convenience sampling method, patients who underwent CABG at three Grade-A tertiary hospitals in Urumqi, Xinjiang, between October 2025 and February 2026 were recruited as study participants. Inclusion criteria were as follows (1): patients meeting the surgical indications for CABG and undergoing the procedure for the first time; (2) aged ≥18 years; (3) hemodynamically stable postoperatively, with no angina pectoris or infective endocarditis. Exclusion criteria were as follows: (1) patients with vital organ insufficiency or terminal illness; (2) patients with a history of mental disorders or severe consciousness, auditory, or speech impairments who were unable to cooperate with the study. This study was approved by the Ethics Committee of the People’s Hospital of Xinjiang Uygur Autonomous Region (Approval No. KY2025102601), and all patients provided written informed consent.

### 2.2 Data collection

Two investigators who received standardized training explained the study objectives and confidentiality principles to the patients in detail. Upon obtaining informed consent, general demographic and clinical data were collected at admission. Questionnaire surveys were conducted on the day of discharge (T1), and at 1 month (T2) and 3 months (T3) post-discharge. After the investigators explained the completion instructions and precautions, patients completed the questionnaires themselves. For those unable to do so due to illness or low literacy, the investigators patiently read each item and recorded the responses on their behalf. A total of 355 patients undergoing CABG were enrolled in this study. During the T2 and T3 follow-up phases, patients attending outpatient visits completed paper questionnaires on-site, while others were surveyed by telephone. During follow-up, 25 patients were lost by T2, and a further 20 patients were lost by T3, yielding 310 patients with complete data at all three time points. Among the 45 patients lost to follow-up, 12 died during the follow-up period and were excluded from all analyses (see **Figure 1**). The remaining 33 patients were retained in the analytic sample through multiple imputation, resulting in a final analytic sample of 343 patients for the network analysis.

**Figure 1:**
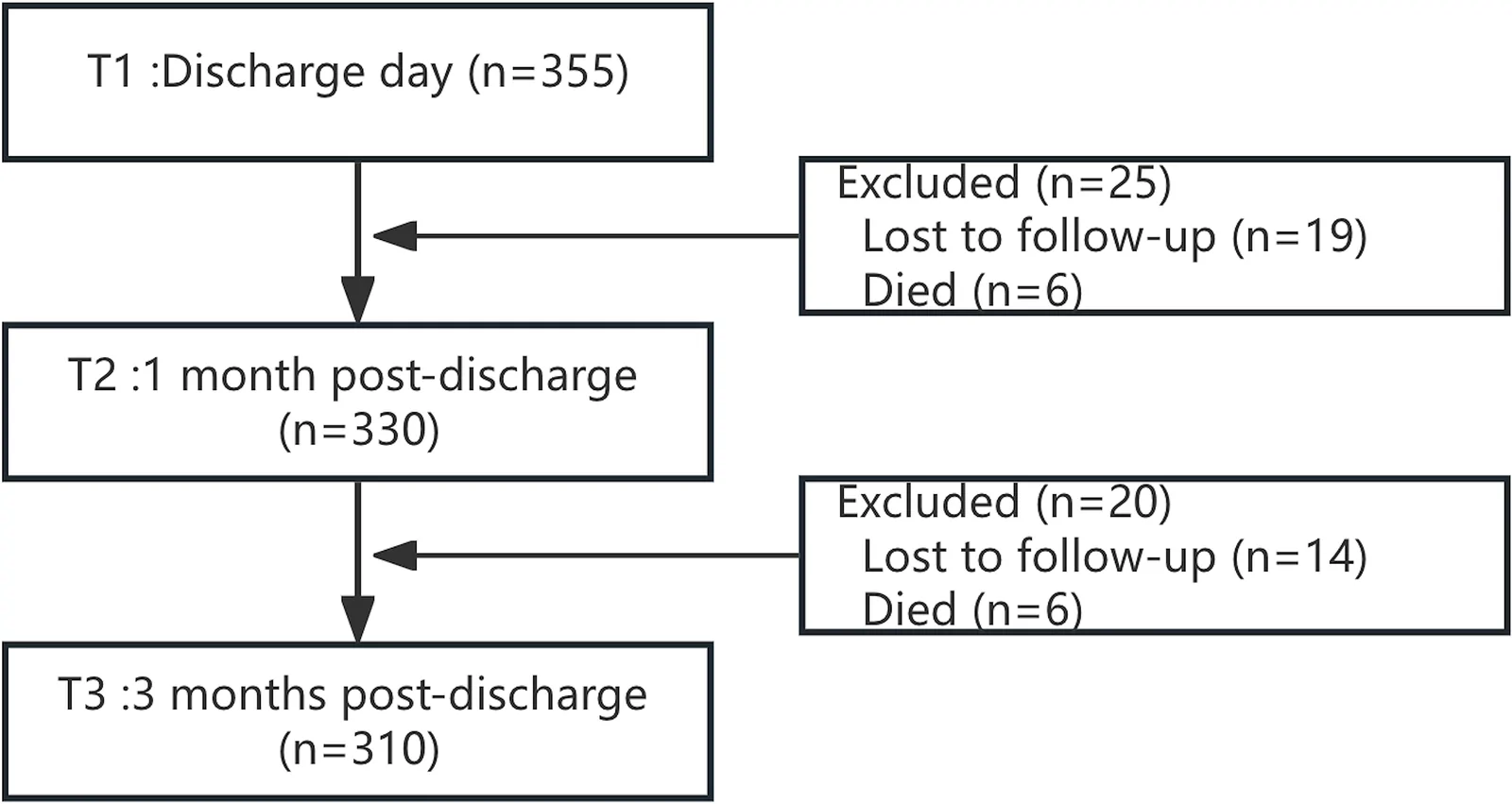
Flow diagram of participant recruitment and exclusion reasons.

### 2.3 Sample size calculation

A parameter recovery evaluation based on Monte Carlo simulations was conducted to assess statistical power and parameter recovery performance under the given sample size (n = 343). The simulation results revealed that the correlation of edge weights, sensitivity, and specificity all exceeded 0.60, indicating that the current sample size provided adequate statistical power and satisfactory robustness for parameter estimation in the cross-lagged panel network analysis (12).

### 2.4 Measurements

#### 2.4.1 Demographic and clinical characteristics

The demographic and clinical characteristics collected in this study included age, gender, educational level, duration of CHD, chronic comorbidities status, and New York Heart Association (NYHA) functional classification.

#### 2.4.2 Generalized anxiety disorder-7, GAD-7

This scale was developed by Spitzer et al. (13). It consists of 7 items, each item scored on a scale from 0 to 3 points, with higher scores indicating more severe anxiety symptoms. In the present study, the Cronbach’s α coefficients for this scale at T1, T2, and T3 were 0.883, 0.829, and 0.718, respectively; the corresponding McDonald’s omega coefficients were 0.915, 0.863, and 0.767, respectively.

#### 2.4.3 Fear of progression questionnaire-short form, FoP-Q-SF

This scale was developed by Mehnert et al. (14). The scale comprises 12 items across two dimensions: physical health and social family, with each item scored on a scale from 1 to 5 points. Higher scores indicate a greater fear of disease progression (15). In the present study, the Cronbach’s α coefficients for the scale at T1, T2, and T3 were 0.889, 0.876, and 0.867, respectively; the corresponding McDonald’s omega coefficients were 0.912, 0.901, and 0.885, respectively.

### 2.5 Statistical analysis

#### 2.5.1 Data preprocessing and missing data imputation

Data analysis was performed using R software (version 4.5.2), with the packages glmnet, qgraph, networktools, ggplot2, bootnet, mgm, dplyr, tidyr, mice, and naniar. For reproducibility, a fixed random seed (12345) was set for all analyses.

First, independent-samples t-tests were conducted to compare differences in study variables between participants who completed the follow-up and those who were lost to follow-up. Given that some patients died during the follow-up period, they were excluded from missing value imputation and subsequent network analyses, resulting in a final sample of 343 patients for network analysis. Furthermore, multiple imputation was employed to address missing data. Five imputations were performed based on the predictive mean matching algorithm (16), and the pooled complete dataset was used for subsequent network analyses.

All demographic data were collected at baseline with no missing values; therefore, these data were not included in the multiple imputation process. The following six variables were identified *a priori* as potential confounders based on prior literature and clinical reasoning: age, gender, educational level, duration of CHD, chronic comorbidities, and NYHA functional classification. These variables were selected because they have been shown to be associated with both anxiety symptoms and fear of progression and, on this basis, may plausibly account for some of the observed symptom network associations (17–22). The two-step residualization method was applied, using multiple linear regression models to partial out the variance associated with the aforementioned covariates from the original symptom variables, and the extracted residuals were then used to construct subsequent network models (23). This residualization procedure removes variance attributable to covariates from symptom scores before network estimation.

#### 2.5.2 Cross-sectional networks

In this study, Gaussian Graphical Models (GGM) were constructed to explore the partial correlation relationships between symptoms at each time point. Given the non-normality of the data distribution, Spearman’s rank correlation was employed for calculation (24). Model selection was performed using the Graphical Least Absolute Shrinkage and Selection Operator (GLASSO) approach combined with the Extended Bayesian Information Criterion (EBIC), with the EBIC hyperparameter gamma set to 0.5. This gamma value represents the standard, widely adopted setting in psychological network analysis literature, balancing model sparsity and sensitivity to true network structure (12, 25). For node centrality assessment, the Expected Influence (EI) index was used to quantify the structural importance of each node within the estimated network. Notably, centrality indices are statistical properties of the network and should not be directly equated with clinical importance.

#### 2.5.3 Cross-lagged panel network analysis

To infer directional longitudinal associations between symptoms over time, a cross-lagged network model was constructed. Node-wise regression with LASSO regularization was employed, with the optimal regularization parameter (lambda) selected separately for each outcome node via 10-fold cross-validation using the minimum cross-validation error criterion (lambda.min). The reported cross-lagged coefficients are LASSO-regularised shrinkage estimates and are not directly comparable to ordinary least squares estimates. The selected lambda value for each outcome node in each network is provided in **Supplementary Table S1**. In the visualized network, nodes represent symptom variables; directed arrows indicate the direction of cross-lagged effects; blue and red edges represent positive and negative associations, respectively; and edge thickness reflects the absolute effect strength. To focus on cross-temporal interactions between symptoms, autoregressive paths were omitted from the network visualization (26). The In-Expected Influence (IEI) and Out-Expected Influence (OEI) were calculated to quantify the extent to which each node is affected by other nodes and its capacity to affect other nodes, respectively. These analyses indicate cross-lagged longitudinal associations, not definitive causality.

#### 2.5.4 Network stability and accuracy

The 95% confidence intervals (CIs) for edge weights were calculated using a nonparametric bootstrap method to assess the accuracy of edge-connection estimates (27). The Correlation Stability (CS) coefficient for centrality indices was calculated using a case-dropping bootstrap method (28). A CS-coefficient > 0.25 indicates acceptable stability, while > 0.50 indicates good stability (29). Furthermore, to formally test whether the observed differences in centrality indices between nodes were statistically significant, bootstrap-based centrality difference tests were performed.

#### 2.5.5 Sensitivity analysis

To evaluate the robustness of findings derived from multiple imputation, we reperformed all network analyses in the original complete case sample (n  =  310). Pearson correlation coefficients were calculated to quantify the agreement between the multiple imputed sample and the original complete case sample for cross-sectional edge weight matrices, EI vectors, as well as cross-lagged edge weight matrices, OEI vectors, and IEI vectors.

## 3 Results

### 3.1 Demographic and clinical characteristics

A total of 355 patients undergoing CABG were enrolled in this study. Demographic and clinical characteristics are presented in **Table 1**. No statistically significant differences were observed in study variables between participants who completed the follow-up and those who were lost to follow-up (*p* > 0.05). Although this comparison provides some indirect support for the plausibility of the missing-at-random (MAR) assumption, it cannot confirm it. Across the three follow-up time points, total GAD-7 scores decreased significantly (6.59 ± 3.92, 4.28 ± 2.99, and 3.41 ± 2.19 at T1, T2, and T3, respectively; repeated-measures ANOVA: F = 177.93, *p* < 0.05, η² = 0.156), while total FoP-Q-SF scores increased significantly (28.32 ± 8.74, 31.34 ± 9.03, and 33.08 ± 8.09 at T1, T2, and T3, respectively; repeated-measures ANOVA: F = 54.33, *p* < 0.05, η² = 0.049). *Post-hoc* comparisons confirmed significant differences between all adjacent time points (all *p* < 0.05). **Table 2** presents item-level information for anxiety and fear of progression nodes.

**Table 1:** Demographic and clinical characteristics of enrolled patients undergoing CABG (n = 355).

Variable	Categories	N (%)/mean ± SD
Age (years)		56.65 ± 10.09
Gender		
	Men	279(78.59)
	Women	76(21.41)
Education (years)		
	≤9	232(65.35)
	>9	123(34.65)
Duration of CHD (years)		
	<2	196(55.21)
	2-5	91(25.63)
	≥5	68(19.16)
Chronic comorbidity status		
	<2	222(62.54)
	≥2	133(37.46)
NYHA functional classification		
	II	173(48.73)
	III/IV	182(51.27)

**Table 2:** The items’ information for anxiety and fear of progression nodes.

Label	Item	T1		T2		T3	
		*M*	*SD*	*M*	*SD*	*M*	*SD*
GAD1	Feeling nervous, anxious or on edge	1.17	0.82	0.53	0.61	0.40	0.51
GAD2	Not being able to stop or control worrying	1.15	0.75	1.15	0.73	0.94	0.62
GAD3	Worrying too much about different things	1.02	0.72	0.99	0.68	0.81	0.66
GAD4	Trouble relaxing	0.89	0.68	0.47	0.60	0.35	0.49
GAD5	Being so restless that it is hard to sit still	0.87	0.69	0.43	0.60	0.36	0.52
GAD6	Becoming easily annoyed or irritable	0.95	0.82	0.48	0.63	0.42	0.53
GAD7	Feeling afraid as if something awful might happen	0.54	0.61	0.23	0.49	0.13	0.37
FoP1	Being afraid of disease progression	3.08	1.33	3.13	1.11	2.68	0.80
FoP2	Being nervous prior to doctors’ appointments or periodic examinations	2.47	1.25	2.90	1.31	3.07	1.24
FoP3	Being afraid of pain	2.79	1.08	2.73	1.10	2.79	1.13
FoP4	Being afraid of becoming less productive at work	2.00	1.15	2.21	1.29	2.46	1.18
FoP5	Having physical symptoms, e.g. rapid heartbeat, stomachache, nervousness	2.73	1.02	2.68	1.09	2.86	1.10
FoP6	Being afraid of the possibility that the children could contract disease	1.99	1.03	2.36	1.25	2.84	1.20
FoP7	Being afraid of relying on strangers for activities of daily living	2.10	1.05	2.68	1.32	2.93	1.09
FoP8	Being afraid of no longer being able to pursue hobbies	2.10	1.02	2.52	1.24	2.80	1.13
FoP9	Being afraid of severe medical treatments in course of the illness	2.96	1.17	3.07	1.17	2.78	1.08
FoP10	Worrying that medications could damage the body	2.43	1.04	2.59	1.12	2.70	1.11
FoP11	Worrying about what will become of the family if something should happen to the patient	2.02	0.95	2.37	1.17	2.61	0.96
FoP12	Being afraid of not being able to work anymore	1.65	0.86	2.10	0.93	2.56	1.05

### 3.2 Cross-sectional networks

The cross-sectional network structures and node centrality indices for anxiety and FoP symptoms at T1, T2, and T3 are presented in **Figure 2**. Each of the networks at T1, T2, and T3 comprised 19 symptom nodes and 171 potential association edges, with 105, 111, and 109 non-zero edges, and network densities of 61.40%, 64.91%, and 63.74%, respectively. At T1 and T2, the core node was consistently FoP11 “Worrying about what will become of the family if something should happen to the patient”, with EI values of 1.29 and 1.24, respectively. Whereas at T3, the core node shifted to FoP2, “Being nervous prior to doctors’ appointments or periodic examinations” (EI = 1.14).

**Figure 2:**
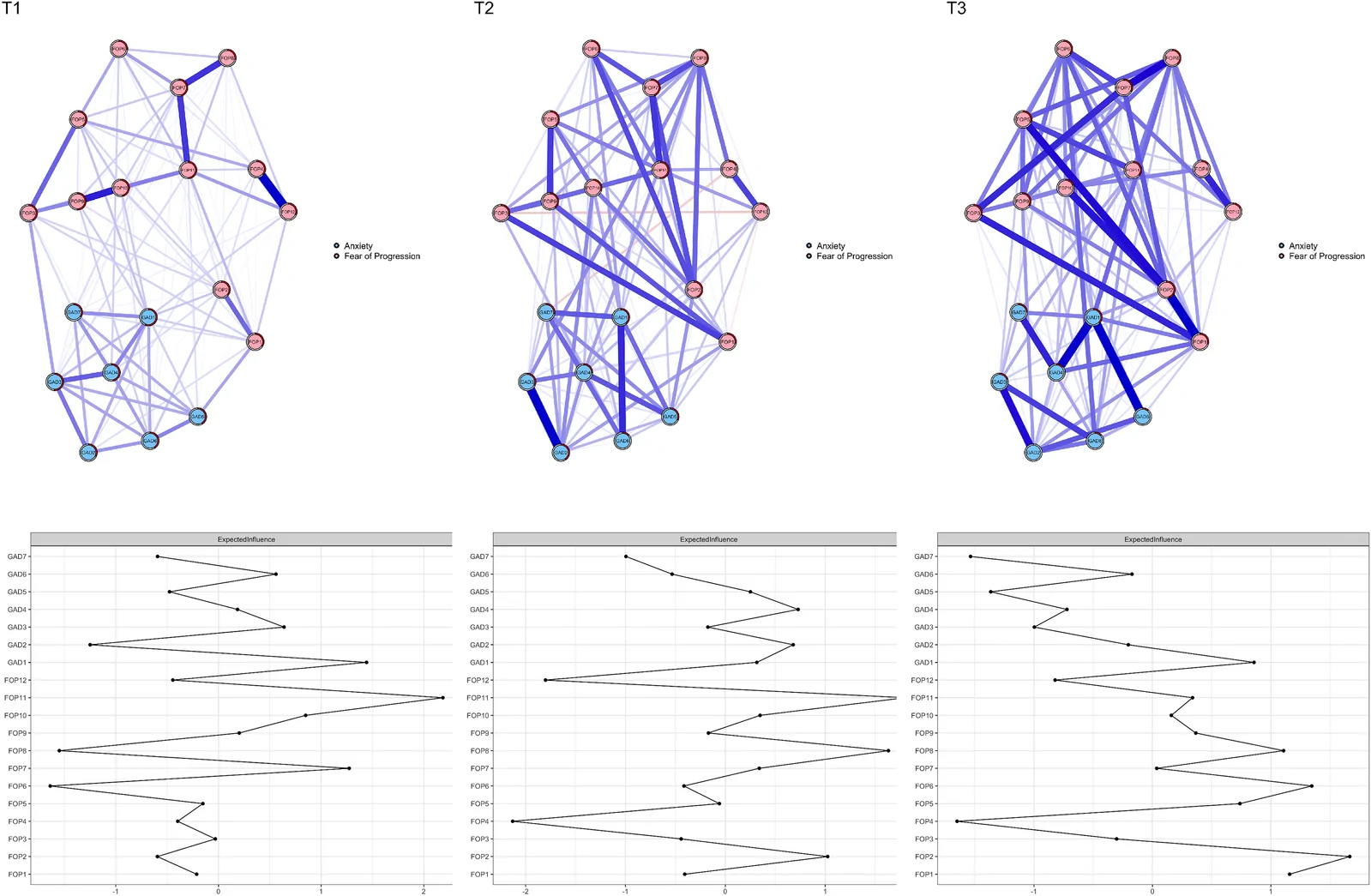
Cross-sectional network structures and centrality indices of anxiety and fear of progression nodes at T1, T2, and T3.

### 3.3 Cross-lagged panel network analysis

The CLPNs for T1-T2, T2-T3, and T1-T3 are shown in **Figure 3**. Each network contained 361 potential association edges. The T1-T2 network identified 196 non-zero edges (54.29%); the T2-T3 network identified 172 non-zero edges (47.65%); and the T1-T3 network identified 143 non-zero edges (39.61%). In the T1-T2 network, the strongest cross-lagged edge was from GAD7 “Feeling afraid as if something awful might happen” to FoP2 “Being nervous prior to doctors’ appointments or periodic examinations” (edge weight = 0.34, 95% bootstrap CI: [0.07, 0.61]). In the T2-T3 network, the strongest cross-lagged edge was from GAD2 “Not being able to stop or control worrying” to FoP6 “Being afraid of the possibility that the children could contract disease” (edge weight = 0.30, 95% bootstrap CI: [0.13, 0.48]). In the T1-T3 network, the strongest cross-lagged edge was from GAD4 “Trouble relaxing” to FoP3 “Being afraid of pain” (edge weight = 0.33, 95% bootstrap CI: [0.10, 0.55]). All three bootstrap confidence intervals excluded zero, indicating that these cross-lagged edges were reliably estimated as non-zero. Because the intervals between time points were unequal, direct comparisons of edge weights across networks should be made with caution.

**Figure 3:**
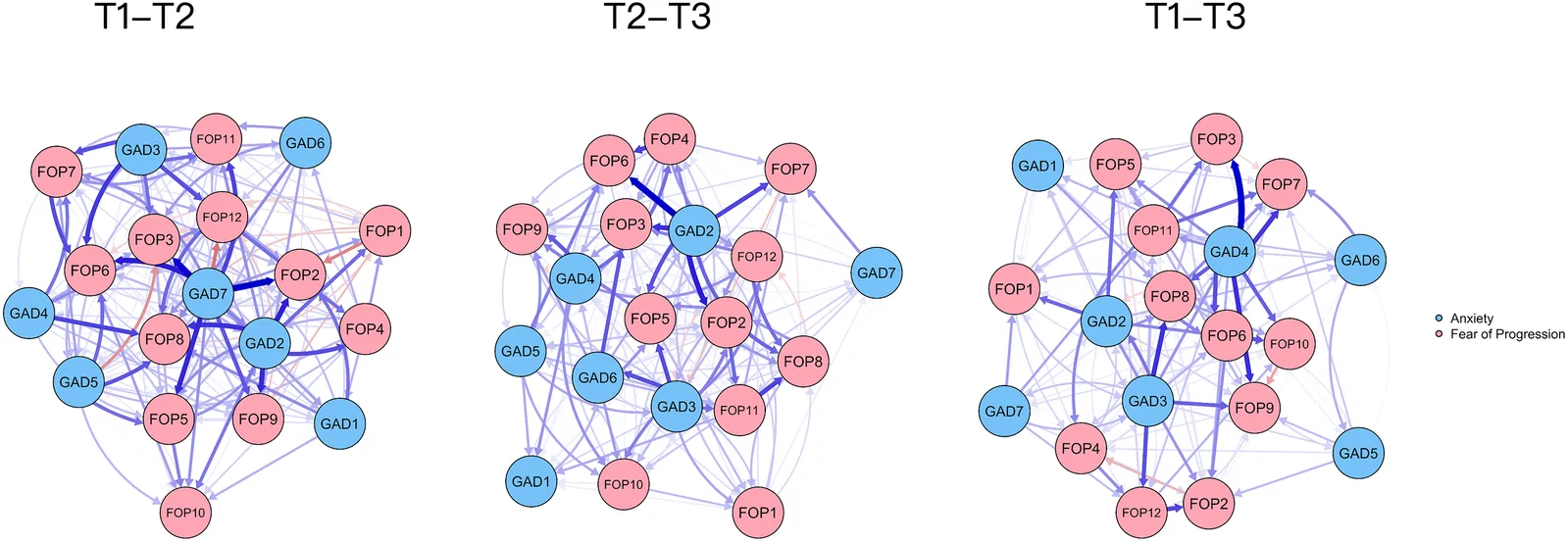
Cross-lagged panel networks of anxiety and fear of progression symptoms across T1-T2, T2-T3, and T1-T3. Blue edges represent positive cross-lagged associations; red edges represent negative cross-lagged associations; edge thickness corresponds to the magnitude of the effect size. Autoregressive paths are omitted for visual clarity.

The centrality of symptoms in the CLPN is depicted in **Figure 4**. In the T1-T2 network, FoP8 “Being afraid of no longer being able to pursue hobbies” showed the highest IEI (IEI = 1.19), while GAD7 “Feeling afraid as if something awful might happen” showed the highest OEI (OEI = 2.21). In the T2-T3 network, FoP2 “Being nervous prior to doctors’ appointments or periodic examinations” showed the highest IEI (IEI = 0.87), while GAD2 “Not being able to stop or control worrying” showed the highest OEI (OEI = 1.69). In the T1-T3 network, FoP7 “Being afraid of relying on strangers for activities of daily living” showed the highest IEI (IEI = 0.75), while GAD4 “Trouble relaxing” showed the highest OEI (OEI = 1.84).

**Figure 4:**
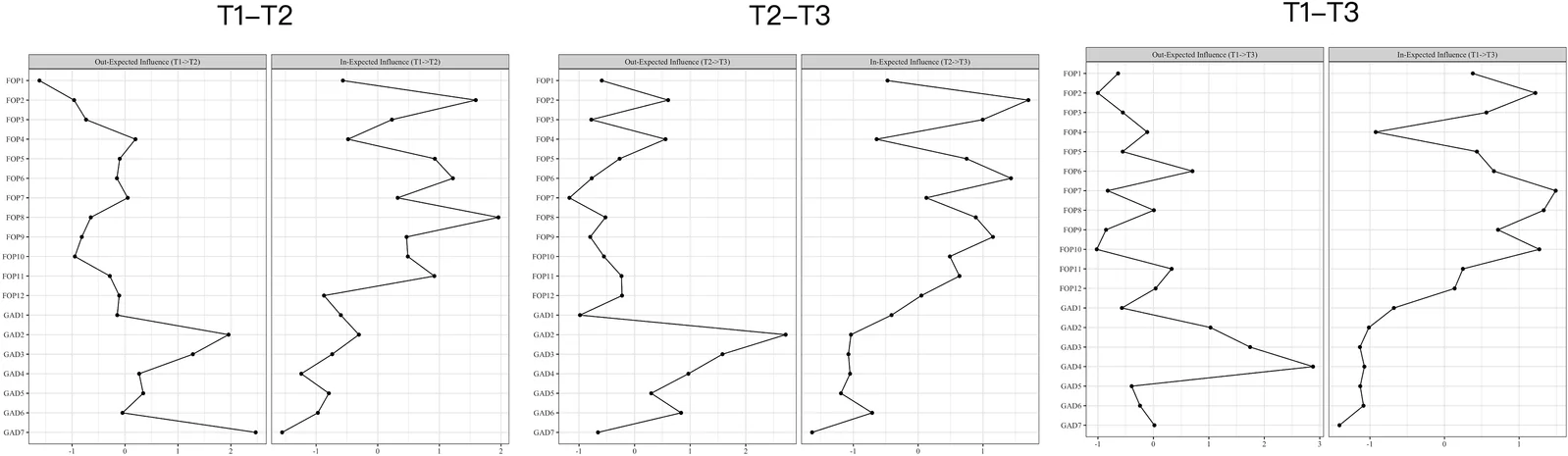
Nodes’ centrality index in the T1-T2, T2-T3, and T1-T3 networks.

### 3.4 Stability and accuracy analysis

The 95% CIs for edge weights in both the cross-sectional network and CLPN were estimated using a non-parametric bootstrap method. The results revealed that the 95% CIs for the edge weights were reasonably narrow, indicating acceptable precision of the edge-weight estimates (**Supplementary Figures 1**, **2**). The CS-coefficients for centrality indices were calculated using a case-dropping bootstrap method. For the cross-sectional networks at T1, T2, and T3, the CS-coefficients for EI were 0.60, 0.44, and 0.21, respectively. For the CLPN, the CS-coefficients for IEI and OEI were 0.67 and 0.36 at the T1-T2 network, 0.52 and 0.28 at the T2-T3 network, and 0.52 and 0.44 at the T1-T3 network, respectively. The majority of CS-coefficients exceeded the commonly recommended threshold of 0.25, indicating acceptable stability of the centrality estimates. The T3 cross-sectional network yielded a slightly lower CS-coefficient (0.21) for EI, suggesting that the centrality rankings at this time point should be interpreted with appropriate caution. To further evaluate whether the differences in centrality indices were statistically significant, bootstrap-based centrality difference tests were conducted (**Supplementary Figures 3**, **4**). Black cells indicate statistically significant differences between two nodes, whereas gray cells indicate non-significant differences. In the cross-sectional networks, FoP11 at T1 and T2 and FoP2 at T3 exhibited high EI, with significantly higher EI than several of the other nodes. In the CLPNs, GAD7 (T1-T2), GAD2 (T2-T3), and GAD4 (T1-T3) exhibited high OEI, and FoP8 (T1-T2), FoP2 (T2-T3), and FoP7 (T1-T3) exhibited high IEI; these nodes likewise showed statistically significantly higher centrality values than several of the other nodes.

### 3.5 Sensitivity analysis

Sensitivity analyses revealed high agreement of cross-sectional edge weights between the two samples (T1 r  =  0.98, T2 r  =  0.98, T3 r  =  0.99). Vectors of EI also showed high consistency (T1 r  =  0.99, T2 r  =  0.97, T3 r  =  0.99). For CLPNs, Pearson correlations of cross-lagged edge weight matrices were r  =  0.90 (T1-T2), r  =  0.97 (T2-T3), and r  =  0.97 (T1-T3). Correlations for OEI vectors were 0.95, 0.98, 0.99; correlations for IEI vectors were 0.97, 0.99, 0.97. Taken together, these high correlation coefficients support the overall robustness of network edge structures and centrality metrics when comparing the multiple imputed sample and the original complete case sample.

## 4 Discussion

This study integrated cross-sectional network analysis with CLPN analysis and conducted longitudinal follow-ups at three critical time points to explore the structural characteristics and reciprocal interactions between anxiety and FoP after CABG, thereby providing empirical evidence to inform the development of phased and precise psychological nursing intervention strategies.

Twelve patients (3.38%) died within three months post-discharge and were excluded from the analyses. This rate is somewhat higher than the 90-day mortality of 1.70% reported by Karim et al. (30); differences in comorbidity burden, age distribution, and healthcare settings may partly account for this discrepancy, although the comparison is descriptive and should be interpreted cautiously given differences in cohort characteristics.

### 4.1 Longitudinal trajectories of anxiety and fear of progression

This study found that the total GAD-7 scores of CABG patients decreased from 6.59 at discharge to 3.41 at 3 months post-discharge, indicating a downward trend in anxiety levels over time, consistent with previous longitudinal studies. For instance, Murphy et al. followed 184 CABG patients for 6 months and observed that 92% of patients with mild preoperative anxiety experienced symptom relief within 6 months postoperatively (31). The early relief of anxiety may be attributed to the alleviation of angina pectoris following successful surgery, the physical benefits derived from improved myocardial perfusion, and the gradual improvement in wound healing and exercise tolerance post-discharge (32). In contrast, total FoP-Q-SF scores rose continuously from 28.32 at T1 to 33.08 at T3, showing an opposite trajectory to anxiety. Conceptually, FoP differs from generalized anxiety: the GAD-7 measures diffuse, domain-unspecific worry and tension, whereas the FoP-Q-SF taps disease-specific, future-oriented fear concerning illness progression and life-threatening complications. These divergent trajectories likely reflect distinct phases of psychological adaptation following CABG. Early-stage anxiety is largely triggered by acute stressors, including surgical trauma, postoperative pain, and uncertainty about immediate clinical outcomes; these stressors abate alongside physical recovery. By comparison, FoP arises from the lifelong nature of CHD management. Despite successful revascularization, patients remain exposed to long-term risks such as graft restenosis, heart failure, and recurrent myocardial infarction. As acute postoperative distress fades, disease-specific worries become the predominant psychological burden, corresponding to a transition from acute illness-related distress to sustained illness vigilance. Collectively, these observations imply that as physical recovery proceeds and anxiety abates after surgery, patients’ concerns about future disease progression intensify (33).

### 4.2 Evolution of centrality indices in cross-sectional networks

At T1 and T2, the core node was FoP11: “Worrying about what will become of the family if something should happen to the patient.” FoP11 reflects patients’ concerns about their family’s well-being if something should happen to them. As rehabilitation proceeds and physical recovery progresses, patients may increasingly contemplate the long-term consequences of their illness for family members, which may partly explain why this family-related concern remained a central node at T1 and T2. By T3, the core node had shifted to FoP2: “Being nervous prior to doctors’ appointments or periodic examinations.” Because CABG improves coronary perfusion without curing CHD, patients still face lifelong disease management demands (34); in this context, routine follow-up visits may serve as reminders of the ongoing cardiac condition and become an increasingly important source of psychological burden, which may explain why this visit-related concern became the central node at T3. This evolutionary trend indicates that core symptoms vary substantially across rehabilitation stages, highlighting the necessity for dynamically adjusted, time-specific intervention strategies (35). It should be emphasized that the core node identified in this study refers to the symptom with the highest centrality index within the estimated network; because centrality is a statistical feature of network modeling rather than direct clinical significance, the clinical value of these core nodes awaits further validation via intervention or experimental studies. In addition, the stability of the centrality estimates at T3 was limited (CS-coefficient = 0.21, below the recommended 0.25 threshold); therefore, the T3 core-node finding should be interpreted with caution.

### 4.3 Temporal relationships in cross-lagged panel networks

Compared with cross-sectional networks, which reflect only static associations, CLPN can capture cross-temporal associations and reciprocal interactions among symptoms through directed network structures (36). It is important to note that, while CLPN estimates temporal lead-lag relationships among observed variables, it cannot establish causal relationships between symptoms. This study found that anxiety nodes generally exhibited higher OEI. In contrast, FoP nodes generally possessed higher IEI, suggesting that anxiety may be prospectively associated with the subsequent development of FoP in CABG patients over the 3-month observation window. These results are consistent with the findings from a CLPN analysis by Chen et al. on breast cancer patients, which reported that death anxiety may be associated with the subsequent development of long-term fear of recurrence (11). According to the cognitive-behavioral model of health anxiety, individuals with elevated anxiety tend to selectively attend to bodily sensations and interpret ambiguous somatic cues as signs of serious disease, a process reinforced by catastrophic misinterpretation and avoidance behaviors (37). CABG patients are continuously subjected to multiple stressors postoperatively, such as graft restenosis, adverse cardiovascular events, and uncertainty regarding long-term prognosis, making them susceptible to generalized anxiety. Anxiety may amplify patients’ negative prognostic judgments regarding disease deterioration (38), which in turn may contribute to the maintenance of persistent FoP over time. However, given the observational nature of this study and the reliance on self-report measures at three discrete time points, these findings should be interpreted as predictive longitudinal associations rather than evidence of a demonstrated causal chain.

In the T1-T2 network, the node with the highest IEI was FoP8 “Being afraid of no longer being able to pursue hobbies.” In the T2-T3 network, the node with the highest IEI was FoP2 “Being nervous prior to doctors’ appointments or periodic examinations.” In the T1-T3 network, the node with the highest IEI was FoP7, “Being afraid of relying on strangers for activities of daily living.” These nodes exhibited high IEI, indicating that their severity at later time points was more strongly associated with earlier symptoms of other nodes (39). They may therefore serve as candidate indicators for monitoring intervention effects, although this requires validation in future intervention or experimental studies.

In the T1-T2 network, GAD7 “Feeling afraid as if something awful might happen” exhibited the highest OEI and showed a strong positive association with FoP2 “ Being nervous prior to doctors’ appointments or periodic examinations.” This suggests that patients who experienced intense fear at discharge were statistically more likely to feel nervous about upcoming medical visits during the first month of home recovery. During the early post-discharge period, patients transition from hospital to home and must interpret bodily symptoms without immediate medical reassurance; in this context, scheduled follow-up visits may serve as reminders of their cardiac condition, potentially triggering anticipatory anxiety. This is consistent with previous reports that elevated anxiety in cardiac patients is associated with heightened symptom vigilance and apprehension about medical encounters (40). Therefore, clinicians and nurses are recommended to deliver brief psychoeducation using written symptom-warning cards that distinguish benign postoperative somatic sensations from red-flag cardiac events, alongside pre-discharge briefings on upcoming follow-up examinations to mitigate anticipatory fear. Furthermore, positive-psychological interventions based on the PERMA model have been shown to reduce FoP and improve subjective well-being among post-CABG patients, offering a valuable theoretical framework for postoperative mental-health care (32).

In the T2-T3 network, GAD2 “Not being able to stop or control worrying” exhibited the highest OEI and showed a strong positive association with FoP6 “Being afraid of the possibility that the children could contract disease.” During home-based rehabilitation, the absence of immediate medical support may heighten patients’ uncertainty regarding long-term cardiovascular risks (34), which may manifest as persistent, intractable excessive worry (41). Such repetitive negative rumination might generalize the risk of their own CHD to family members, possibly contributing to a fear of potential hereditary transmission to their children (42). A large-scale cohort study by Galimberti et al. suggested that CHD possesses a distinct familial genetic predisposition; having even one first-degree relative with the disease significantly increases the risk of coronary atherosclerosis in offspring, and if both parents are affected, the risk nearly doubles (43), which may further intensify patients’ concerns regarding their children’s health. These findings suggest that during home-based follow-up commencing one month post-discharge, clinical attention may reasonably focus on patients with uncontrollable excessive worry. For affected patients, structured family-oriented cognitive counseling may provide plain-language education on familial CHD risk, differentiating genetic susceptibility from inevitable disease onset; family-member participation in consultations may be considered when clinically appropriate to assist patients in recognizing and reframing catastrophic disease cognitions.

In the T1-T3 network, GAD4 “Trouble relaxing” exhibited the highest OEI, and its strong positive association was likewise with FoP3 “Being afraid of pain.” Due to the trauma of open-heart surgery and prolonged rehabilitation stress leading to sustained activation of the sympathetic nervous system, the excessive secretion of catecholamines may be associated with somatic responses such as muscle tension, elevated blood pressure, and tachycardia, potentially keeping patients in a chronic state of stress characterized by difficulty relaxing (44). In such a chronic tense state, patients may misread routine rehabilitation-related physical symptoms like palpitations, incisional traction pain, and fatigue as indicators of disease progression (45), thereby inducing avoidant behaviors and fear of pain (40). Previous studies have confirmed that deep breathing exercises and progressive muscle relaxation training represent effective, low-cost, nurse-delivered approaches to reduce anxiety in post-CABG patients (46, 47). Accordingly, clinical staff should conduct screening for persistent difficulty relaxing across all early rehabilitation time points. Nurses can administer standardized brief progressive muscle relaxation or diaphragmatic breathing training supplemented with take-home guidance and verify patients’ skill mastery during subsequent follow-up visits.

## 5 Limitations

This study has several limitations. First, convenience sampling was used to recruit participants solely from three Grade-A tertiary hospitals in Urumqi, Xinjiang, a culturally and linguistically diverse region of China. The sample was predominantly male (78.59%), and all participants originated from one geographical setting. These factors may introduce selection bias and restrict the geographical representativeness and generalizability of the present findings. Second, psychological assessments were conducted at only three time points: discharge, 1 month, and 3 months post-discharge. The relatively brief follow-up may fail to capture longer-term psychological trajectories at 6 months, 1 year, or beyond. Additionally, measurement waves featured unequal time lags: approximately one month separated T1 and T2, whereas two months separated T2 and T3. Given that cross-lagged coefficients depend on the length of time lags, effect magnitudes derived from different time windows cannot be directly compared, limiting cross-model comparisons of our CLPN outputs. Future investigations could adopt equal assessment intervals or continuous-time modeling to address this constraint. Furthermore, although CLPN can detect cross-temporal longitudinal associations among symptoms, it cannot establish definitive causal relationships. Hence, the psychological mechanisms proposed to interpret these observed associations remain tentative and require validation in future experimental or intervention studies. It should also be noted that not all stability coefficients met the recommended threshold; specifically, the CS-coefficient based on EI for the T3 cross-sectional network was 0.21, slightly below the 0.25 cutoff, with several other CS-coefficients approaching this threshold. Accordingly, centrality results from these networks need to be interpreted with caution. Third, the internal consistency of the GAD-7 decreased from 0.883 at T1 to 0.718 at T3. Several anxiety items showed near-zero mean values with restricted variances at T3, pointing to potential floor effects. Fourth, while no statistically significant differences were detected between completers and participants lost to follow-up, the MAR assumption cannot be formally verified. Missing data may be associated with unmeasured symptom severity, especially among patients who died or withdrew owing to health deterioration. Sensitivity analyses under missing-not-at-random assumptions, such as pattern mixture models or tipping point analysis, would help evaluate the robustness of our results.

## 6 Conclusion

This study integrates cross-sectional network analysis and CLPN analysis to reveal the longitudinal interaction between anxiety and FoP symptoms among patients in the early rehabilitation stage after CABG. The results demonstrated that patients’ anxiety levels gradually declined after discharge, while their fear of disease progression showed a continuous upward trend. Cross-sectional network analyses demonstrated that the core node from T1 to T2 was FoP11 “Worrying about what will become of the family if something should happen to the patient.” In contrast, the core node shifted to FoP2 “Being nervous prior to doctors’ appointments or periodic examinations, “ at T3. CLPN analysis revealed that anxiety nodes generally yielded higher OEI, while FoP nodes had higher IEI. In the T1-T2 network, GAD7 “Feeling afraid as if something awful might happen” exhibited the highest OEI and was positively associated with subsequent T2 FoP2 “Being nervous prior to doctors’ appointments or periodic examinations.” In the T2-T3 network, GAD2 “Not being able to stop or control worrying” emerged as the node with the maximum OEI, which was positively associated with subsequent T3 FoP6 “Being afraid of the possibility that the children could contract disease.” In the T1-T3 network, GAD4 “Trouble relaxing” had the highest OEI and was positively associated with subsequent T3 FoP3 “Being afraid of pain.” In conclusion, clinical medical staff and nurses should implement phased, precise intervention strategies tailored to the core symptoms of CABG patients across different rehabilitation stages to improve patients’ long-term physical and mental rehabilitation outcomes.

## Data Availability

The raw data supporting the conclusions of this article will be made available by the authors, without undue reservation.
