# Supplementary material for: Longitudinal relationships between anxiety and fear of progression in post-CABG patients: a cross-sectional and cross-lagged panel network analysis
Source: Front Psychiatry. 2026 Oct 14;17:1937200. doi: 10.3389/fpsyt.2026.1937200 (PMC13713660; doi:10.3389/fpsyt.2026.1937200)
Supplement: Supplementary file 1 [file SupplementaryFile1.docx]

Supplementary Material

Table S1 The selected lambda value of the node-wise LASSO regression in each network.

| Node | T1_T2 | T2_T3 | T1_T3 |
| --- | --- | --- | --- |
| GAD1 | 2.602889e-02 | 1.617454e-02 | 2.058250e-02 |
| GAD2 | 1.182706e-02 | 2.844488e-02 | 3.222694e-02 |
| GAD3 | 2.749901e-02 | 1.506142e-02 | 2.795081e-02 |
| GAD4 | 2.369150e-02 | 1.481293e-02 | 2.727310e-02 |
| GAD5 | 1.725802e-02 | 1.659981e-02 | 3.597872e-02 |
| GAD6 | 2.125520e-02 | 2.504239e-02 | 2.463562e-02 |
| GAD7 | 2.665787e-02 | 1.654808e-02 | 2.376950e-02 |
| FOP1 | 4.791404e-02 | 5.042081e-02 | 2.812715e-02 |
| FOP2 | 4.402058e-02 | 5.348493e-02 | 4.206600e-02 |
| FOP3 | 2.594769e-02 | 4.741584e-02 | 9.064691e-02 |
| FOP4 | 6.647709e-02 | 6.348919e-02 | 4.430845e-02 |
| FOP5 | 4.648231e-02 | 2.707072e-02 | 4.659063e-02 |
| FOP6 | 4.524320e-02 | 5.609343e-02 | 6.868872e-02 |
| FOP7 | 4.454152e-02 | 5.635466e-02 | 2.802789e-02 |
| FOP8 | 3.287381e-02 | 5.275335e-02 | 5.296806e-02 |
| FOP9 | 3.461893e-02 | 2.585716e-02 | 3.884653e-02 |
| FOP10 | 4.600732e-02 | 5.922939e-02 | 3.525655e-02 |
| FOP11 | 6.652122e-02 | 2.807852e-02 | 4.467153e-02 |
| FOP12 | 6.448507e-02 | 2.872726e-02 | 5.136004e-02 |

**Supplementary Figure 1** Bootstrapped 95% confidence intervals of edge weights in the cross-sectional networks (T1, T2, and T3).

**Supplementary Figure 2** Bootstrapped 95% confidence intervals of cross-lagged edge weights in the cross-lagged panel networks (T1-T2, T2-T3, and T1-T3).

**Supplementary Figure 3** Centrality difference tests for Expected Influence based on non-parametric bootstrapping in the cross-sectional networks (T1, T2, and T3). Black cells indicate statistically significant differences between centrality indices; gray cells indicate non-significant differences.

**Supplementary Figure 4** Centrality difference tests for Out-Expected Influence and In-Expected Influence based on non-parametric bootstrapping in the cross-lagged panel networks (T1-T2, T2-T3, and T1-T3). Black cells indicate statistically significant differences between centrality indices; gray cells indicate non-significant differences.
